# Quantum Coherence and Purity in Dissipative Hydrogen Atoms: Insights from the Lindblad Master Equation

**DOI:** 10.3390/e27080848

**Published:** 2025-08-10

**Authors:** Kamal Berrada, Smail Bougouffa

**Affiliations:** Department of Physics, College of Science, Imam Mohammad Ibn Saud Islamic University (IMSIU), P.O. Box 90950, Riyadh 11623, Saudi Arabia; sbougouffa@imamu.edu.sa

**Keywords:** hydrogen atoms, Lindblad master equation, coherence, von Neumann entropy, hyperfine Hamiltonian

## Abstract

In this work, we investigate the quantum coherence and purity in hydrogen atoms under dissipative dynamics, with a focus on the hyperfine structure states arising from the electron–proton spin interaction. Using the Lindblad master equation, we model the time evolution of the density matrix of the system, incorporating both the unitary dynamics driven by the hyperfine Hamiltonian and the dissipative effects due to environmental interactions. Quantum coherence is quantified using the $L_{1}$ norm and relative entropy measures, while purity is assessed via von Neumann entropy, for initial states, including a maximally entangled Bell state and a separable state. Our results reveal distinct dynamics: for the Bell states, both coherence and purity decay exponentially with a rate proportional to the dissipation parameter, whereas for a kind of separable state, coherence exhibits oscillatory behavior modulated via the hyperfine coupling constant, superimposed on an exponential decay, and accompanied by a steady increase in entropy. Higher dissipation rates accelerate the loss of coherence and the growth of von Neumann entropy, underscoring the environment’s role in suppressing quantum superposition and driving the system towards mixed states. These findings enhance our understanding of coherence and purity preservation in atomic systems and offer insights for quantum information applications where robustness against dissipation is critical.

## 1. Introduction

The hydrogen atom, with its elegantly simple structure, has historically served as a cornerstone of quantum mechanics understanding, offering profound insights into the behavior of electrons and nuclei in diverse physical, chemical, and biological contexts [1,2,3,4]. Beyond its foundational role in quantum theory, the hydrogen atom emerges as a pivotal element in quantum information science, providing a natural system for exploring quantum correlations. The electron and nuclear spins in the hydrogen atom offer a physically intuitive framework and a well-defined Hilbert space for investigating bipartite quantum entanglement, whose entanglement, quantified by two-qubit concurrence, can be directly linked to fundamental constants such as the Planck constant, the Boltzmann constant, electron and proton masses, the fine-structure constant, the Bohr radius, and the Bohr magneton. At low temperatures, the hyperfine structure (HFS) states of the hydrogen atom exhibit inherent entanglement, which diminishes rapidly as the temperature increases, eventually disappearing beyond a critical threshold of $\tau_{c}\approx 5.35\;\mu \mathrm{eV}$. This phenomenon is rooted in the thermal equilibrium behavior of the HFS states, where the entanglement is sensitive to the balance between the energy gap and thermal energy [5,6,7]. Recent studies have further revealed nuclear-polarized phases of hydrogen atoms embedded in solid H_2_ films [5,8], showcasing significant deviations from the Boltzmann distribution at low temperatures [5,6,7], which raises intriguing questions about the role of quantum effects in such systems. The electron- and nuclear-spin degrees of freedom in the hydrogen atom provide a platform for studying entanglement and connecting to broader applications in quantum information. Earlier research on electron-spin dynamics in two-electron double-quantum-dot systems [9,10] has demonstrated the potential of such systems as qubits for quantum information technologies [11,12,13]. Similarly, nuclear spins, particularly in systems like nitrogen-vacancy centers in diamonds, have been identified as valuable resources for quantum information processing [14,15,16,17]. In contrast to previous works that explored electron–proton coordinate entanglement [18] or provided a formalism for HFS entanglement [19], this study uncovers a novel aspect: the ability of an external magnetic field to induce and sustain HFS entanglement even at temperatures well above the critical threshold $\tau_{c}$. This magnetically induced entanglement defies the thermal degradation typically observed in such systems, offering a new avenue for entanglement engineering in low-temperature environments, including gases and solids.

Quantum coherence (QC), a phenomenon emerging from the superposition principle, constitutes a cornerstone of quantum mechanics and serves as an essential resource across a spectrum of quantum information processing applications, including quantum reference frames [20,21,22], quantum transport within biological systems [23,24,25], and quantum thermodynamics [26,27,28]. The task of quantifying QC represents a critical challenge both in the theoretical foundations of quantum mechanics and in the practical domain of quantum information science, drawing significant research focus in recent years [29]. This framework has notably elucidated the role of coherence in underpinning quantum advantages, such as quantum state merging [30], deterministic quantum computation with one qubit [31], the Deutsch–Jozsa algorithm [32], and Grover’s search algorithm [33]. Furthermore, the resource theory of coherence provides a robust foundation for interpreting the wave-like characteristics of quantum systems [34], as well as the intrinsic nature of quantum correlations, encompassing entanglement [35] and an array of discord-like measures [36]. Baumgratz et al. [29] have recently advanced a resource-theoretic framework to systematically quantify QC in quantum states, paving the way for the development of diverse coherence measures rooted in distinct physical principles. The initial proposals included the norm of coherence and the relative entropy of coherence, both based on distance-based metrics [29]. This was followed by subsequent measures that leveraged entanglement [37], operational perspectives [38,39], and convex-roof constructions [40,41]. These quantitative tools have enabled detailed investigations into QC’s multifaceted properties, such as its interconnections with other quantum resources [37,42,43], its manifestation in infinite-dimensional Hilbert spaces [44,45], its complementarity relations [46], and the quantification of macroscopic coherence [47]. This resource-theoretic approach to QC quantification has thus catalyzed a broad array of further explorations into the nature and implications of QC [48,49,50,51].

The theory of open quantum systems examines the dynamics of quantum systems that interact with their surrounding environments, a topic of significant interest since the foundational development of quantum mechanics [52]. Despite considerable theoretical progress, fundamental challenges remain unresolved, notably the phenomenon of decoherence, which involves the loss of quantum coherence due to interactions between a system and its environment.

Recent progress has considerably deepened our understanding of quantum coherence, particularly its behavior in decoherence and noisy environments. Key studies have explored phenomena such as frozen coherence, analytical evolution equations, noise coherence-generating power, and coherence dynamics in correlated channels [53,54,55,56]. These contributions provide essential tools and perspectives for analyzing coherence loss and preservation in open quantum systems.

Building on these developments, it is clear that understanding coherence dynamics is essential not only from a fundamental perspective but also for practical uses. This process has attracted substantial attention in the fields of quantum information and computation, where decoherence remains one of the central challenges in the realization of scalable quantum information processors [57,58,59]. The preservation of QC is indispensable for the operation of quantum computers, quantum cryptography, and quantum teleportation. Furthermore, decoherence serves as a critical mechanism for understanding the quantum-to-classical transition, wherein the emergence of classical properties from quantum systems is interpreted as a consequence of environmentally induced decoherence. In this study, we investigate the QC and purity in hydrogen atoms under dissipative dynamics using the Lindblad master equation. We will show that Bell states exhibit exponential decay in both coherence and purity, while separable states show oscillatory coherence with decay and rising entropy, effects that worsen with higher dissipation rates. The results obtained highlight the role of the environment in the degradation of the QC and purity, providing critical insights for the preservation of quantum properties in atomic systems.

This manuscript is structured as follows. Section 2 describes the system Hamiltonian and presents the solution to the physical model. In Section 3, we introduce the coherence measure and analyze its time-dependent behavior for the model under consideration. Finally, Section 4 summarizes the key findings of this study.

## 2. The Hamiltonian and Quantum Dynamics

The ground state of the hydrogen atom exhibits a fascinating interplay of spin degrees of freedom governed by the hyperfine interaction Hamiltonian. This Hamiltonian captures the magnetic dipole–dipole coupling between the electron and proton spins and is mathematically expressed as

(1) $$H_{\mathrm{HF}}=A\left(\sigma_{e}\cdot \sigma_{p}\right).$$

Here, $\sigma_{e}=\left(\sigma_{e}^{x},\sigma_{e}^{y},\sigma_{e}^{z}\right)$ represents the vector of Pauli operators for the electron spin, while $\sigma_{p}=\left(\sigma_{p}^{x},\sigma_{p}^{y},\sigma_{p}^{z}\right)$ denotes the corresponding vector for the proton spin. These operators act on the spin-1/2 nature of both particles, encoding their quantum mechanical spin properties along the three spatial axes. The parameter *A*, termed the hyperfine structure constant, quantifies the strength of this interaction. It arises from a combination of fundamental physical constants, including the fine-structure constant α, which governs electromagnetic interactions; the electron and proton *g*-factors, $g_{e}$ and $g_{p}$, which adjust the magnetic moments relative to the Bohr magneton and nuclear magneton; the vacuum permeability $\mu_{0}$; the reduced Planck constant *ℏ*; the Bohr radius $a_{0}$, a measure of the electron–proton separation in the ground state; and the masses of the electron $m_{e}$ and proton $m_{p}$. The precise form of this constant is given by $A=\frac{8}{3}\alpha^{2}g_{e}g_{p}\frac{\mu_{0}}{4\pi }\frac{\hbar^{2}}{a_{0}^{3}}\frac{m_{e}}{m_{p}}$. This hyperfine Hamiltonian dictates the energy level structure of the hydrogen atom’s ground state by coupling the electron and proton spins into a total spin system. Given that both the electron and the proton are spin-1/2 particles, their spins can either align to form a triplet configuration with total spin $S_{\mathrm{total}}=1$ or anti-align to form a singlet configuration with $S_{\mathrm{total}}=0$. The eigenvalues of $H_{\mathrm{HF}}$ reveal this splitting: the triplet states, characterized by parallel spin orientations, possess an energy of E=A, whereas the singlet state, with antiparallel spins, has an energy of E=−3A. The energy separation between these levels, known as hyperfine splitting, is thus ΔE=4A.

The effective Hamiltonian in Equation (1) describes the hyperfine interaction in the hydrogen atom, valid when the electron occupies the ground (1s) state. In this regime, the dominant spin-dependent interaction is the magnetic dipole–dipole coupling between the electron and proton spins. The Coulomb binding and spin–orbit coupling are either already accounted for in the energy level structure or negligible (e.g., spin–orbit vanishes for l=0).

The Hamiltonian eigenstates span a four-dimensional Hilbert space given by the computational basis $B={\{|↑}_{e}{↑}_{p}{\rangle ,|↑}_{e}{↓}_{p}{\rangle ,|↓}_{e}{↑}_{p}{\rangle ,|↓}_{e}{↓}_{p}\rangle \}.$ Here, |↑〉 and |↓〉 denote spin-up and spin-down states along the *z*-axis for the electron (subscript *e*) and proton (subscript *p*). The energy eigenvalues are derived using the total spin operator $S=S_{e}+S_{p}$. For triplet states ($S_{\mathrm{total}}=1$), $S^{2}=2\hbar^{2}$, giving energy E=A; for the singlet state ($S_{\mathrm{total}}=0$), $S^{2}=0$, giving E=−3A. The singlet eigenstate, |a〉, is a Bell state:

(2) $$|a\rangle =\frac{1}{\sqrt{2}}\left({|↑}_{e}{↓}_{p}{\rangle -|↓}_{e}{↑}_{p}\rangle \right),$$

with energy $E_{a}=-3A$. The triplet eigenstates (E=A) include two separable states:

(3) $$|d\rangle ={|↑}_{e}{↑}_{p}\rangle ,$$

(4) $$|b\rangle ={|↓}_{e}{↓}_{p}\rangle ,$$

with energies $E_{d}=E_{b}=A$, representing spins aligned up or down. The third triplet state, |c〉, is another entangled Bell state:

(5) $$|c\rangle =\frac{1}{\sqrt{2}}\left({|↑}_{e}{↓}_{p}{\rangle +|↓}_{e}{↑}_{p}\rangle \right),$$

with energy $E_{c}=A$. The presence of entangled eigenstates such as |a〉 and |c〉 within the hyperfine structure illustrates the hydrogen atom’s intrinsic quantum mechanical richness. These states, alongside the separable triplet states |b〉 and |d〉, fully characterize the spin dynamics of the system in its ground state. The energy difference ΔE=4A drives observable phenomena, such as the 21 cm spectral line. This structure not only provides insight into atomic physics but also serves as a foundational example for understanding quantum correlations in more complex systems, enabling studies of QC and von Neumann dynamics under dissipation, as explored in this work.

The system is characterized by the hyperfine Hamiltonian and dissipative interactions modeled via Lindblad operators representing spin-flip processes for the electron and proton. The time evolution of the density matrix ρ(t) is governed via the Lindblad master equation, which accounts for both unitary evolution and dissipation

(6) $$\frac{d\rho (t)}{dt}=-i\left[H_{\mathrm{HF}},\rho \left(t\right)\right]+D\left(\rho \left(t\right)\right),$$

where D(ρ(t)) is the dissipation superoperator. The dissipative term is expressed as

(7) $$D\left(\rho \right)=\frac{\gamma }{2}\sum_{k=1}^{4}\left(L_{k}\rho L_{k}^{†}-\frac{1}{2}\left\{L_{k}^{†}L_{k},\rho \right\}\right),$$

where γ represents the dissipation rate, and $L_{k}$ are the Lindblad operators describing spin-flip processes. These operators are defined as

(8) $$\begin{array}{cccc} L_{1} & =\sigma_{+}^{e}⊗I_{p}, & \; & (\mathrm{electron}\;\mathrm{spin}\text{-}\mathrm{up}\;\mathrm{transition}) \end{array}$$

(9) $$\begin{array}{cccc} L_{2} & =\sigma_{-}^{e}⊗I_{p}, & \; & (\mathrm{electron}\;\mathrm{spin}\text{-}\mathrm{down}\;\mathrm{transition}) \end{array}$$

(10) $$\begin{array}{cccc} L_{3} & =I_{e}⊗\sigma_{+}^{p}, & \; & (\mathrm{proton}\;\mathrm{spin}\text{-}\mathrm{up}\;\mathrm{transition}) \end{array}$$

(11) $$\begin{array}{cccc} L_{4} & =I_{e}⊗\sigma_{-}^{p}, & \; & (\mathrm{proton}\;\mathrm{spin}\text{-}\mathrm{down}\;\mathrm{transition}) \end{array}$$

Here, $\sigma_{+}=\left|↑\rangle \langle ↓\right|$ and $\sigma_{-}=\left|↓\rangle \langle ↑\right|$ denote the raising and lowering operators for the respective spin systems. Further, we assume equal rates for the excitation and decay processes, corresponding to the high-temperature limit of a thermal reservoir, where $\bar{n}\to \infty$ and Γ→0, such that the product $\Gamma \bar{n}\equiv \gamma$ remains finite. This approximation leads to symmetric noise channels and is consistent with the infinite-temperature limit described in Ref. [60] (see Equation (112) and its discussion). Although we adopt equal rates for simplicity, the model can be extended to include temperature-dependent asymmetric rates, which will be explored in future work.

The time derivative $\frac{d\rho }{dt}$ yields a set of coupled differential equations for the matrix elements $\rho_{ij}\left(t\right)$, where i,j=1,2,3,4 correspond to the computational basis. These equations account for coherent interactions, parameterized by the coupling strength *A*, and dissipative processes, characterized by the dissipation rate γ. The equations are separated into those governing the diagonal elements (populations) and the off-diagonal elements (coherences) for clarity.

The diagonal elements $\rho_{ii}\left(t\right)$ represent the probabilities of finding the system in each basis state. Their time evolution is given as follows:

(12) $$\begin{array}{cc} \frac{d\rho_{11}}{dt} & =\gamma (\rho_{22}+\rho_{33}-2\rho_{11}), \end{array}$$

(13) $$\begin{array}{cc} \frac{d\rho_{22}}{dt} & =-2iA\left(\rho_{23}-\rho_{32}\right)+\gamma \left(\rho_{11}+\rho_{44}-2\rho_{22}\right), \end{array}$$

(14) $$\begin{array}{cc} \frac{d\rho_{33}}{dt} & =2iA\left(\rho_{23}-\rho_{32}\right)+\gamma \left(\rho_{11}+\rho_{44}-2\rho_{33}\right), \end{array}$$

(15) $$\begin{array}{cc} \frac{d\rho_{44}}{dt} & =\gamma (\rho_{22}+\rho_{33}-2\rho_{44}). \end{array}$$

Here, the terms involving γ describe population transfer due to dissipation, while the terms with *A* in $\rho_{22}$ and $\rho_{33}$ arise from coherent coupling between states ${|↑}_{e}{↓}_{p}\rangle$ and ${|↓}_{e}{↑}_{p}\rangle$.

The off-diagonal elements $\rho_{ij}\left(t\right)$ (for i≠j) represent the coherence between the basis states. Their dynamics are governed via the following:

(16) $$\begin{array}{cc} \frac{d\rho_{12}}{dt} & =-2iA\left(\rho_{12}-\rho_{13}\right)+\gamma \left(\rho_{34}-2\rho_{12}\right), \end{array}$$

(17) $$\begin{array}{cc} \frac{d\rho_{13}}{dt} & =-2iA\left(\rho_{13}-\rho_{12}\right)+\gamma \left(\rho_{24}-2\rho_{13}\right), \end{array}$$

(18) $$\begin{array}{cc} \frac{d\rho_{14}}{dt} & =-2\gamma \rho_{14}, \end{array}$$

(19) $$\begin{array}{cc} \frac{d\rho_{23}}{dt} & =-2iA\left(\rho_{22}-\rho_{33}+\rho_{23}\right)-2\gamma \rho_{23}, \end{array}$$

(20) $$\begin{array}{cc} \frac{d\rho_{24}}{dt} & =-2iA\left(\rho_{24}-\rho_{34}\right)+\gamma \left(\rho_{13}-2\rho_{24}\right), \end{array}$$

(21) $$\begin{array}{cc} \frac{d\rho_{34}}{dt} & =-2iA\left(\rho_{24}-\rho_{34}\right)+\gamma \left(\rho_{12}-2\rho_{34}\right). \end{array}$$

These equations include both coherent evolution (terms with *A*) and decoherence (terms with γ). In particular, $\rho_{14}$ decays purely dissipatively, indicating that there is no coherent coupling between states ${|↑}_{e}{↑}_{p}\rangle$ and ${|↓}_{e}{↓}_{p}\rangle$. The remaining off-diagonal elements ($\rho_{21}$, $\rho_{31}$, $\rho_{32}$, $\rho_{41}$, $\rho_{42}$, $\rho_{43}$) are determined by Hermitian conjugation, ensuring the density matrix remains Hermitian ($\rho^{†}=\rho$). Specifically, $\rho_{ji}=\rho_{ij}^{*}$ for all i,j. These differential equations fully describe the dynamics of the system under the combined effects of coherent interactions and dissipation. They can be solved analytically under specific initial conditions.

## 3. Quantum Coherence and Results

In the resource-theoretic framework of QC, the $l_{1}$-norm of coherence serves as a fundamental measure to quantify the coherence present in a quantum state, is given by [29]

(22) $$C_{l_{1}}\left(\rho \right)=\sum_{i\neq j}\left|\rho_{i,j}\right|,$$

where ρ is the density matrix, and the sum captures the absolute values of its off-diagonal elements. This metric provides a straightforward and computationally efficient way to assess the magnitude of quantum superpositions, making it particularly valuable for analyzing coherence dynamics in systems like hydrogen atoms under dissipative conditions, as explored in this manuscript. Complementing this, the relative entropy of coherence, given by

(23) $$C_{\mathrm{rel}}\left(\rho \right)=S\left(\rho_{\mathrm{diag}}\right)-S\left(\rho \right),$$

where *S* denotes the von Neumann entropy, and $\rho_{\mathrm{diag}}$ is the diagonal part of ρ, which measures coherence through the entropic difference between the incoherent (diagonal) state and the full quantum state. This information-theoretic measure effectively captures the quantum information encoded in off-diagonal terms and adheres to essential properties such as vanishing for incoherent states, monotonicity under incoherent operations, and convexity. Both measures align with the resource theory of coherence, ensuring that coherence remains a non-increasing resource under free (incoherent) operations.

Contemporary studies have shown that entropy generation within the framework of classical thermodynamics can be understood as the development of correlations between an open quantum system and its surrounding environment [61,62,63,64]. For a quantum state, ρ, representing a qubit, the von Neumann entropy is defined by the expression

(24) S(ρ)=−tr(ρlnρ).

In the context of a bipartite quantum system, the entropies of the individual subsystems and the combined system conform to the inequalities

(25) $$\left|S\left(\rho_{A}\right)-S\left(\rho_{B}\right)\right|\leq S\left(\rho_{AB}\right)\leq S\left(\rho_{A}\right)+S\left(\rho_{B}\right),$$

where *A* and *B* designate the two distinct subsystems.

Given the initial state,

(26) $${\cos\alpha |↑}_{e}{↓}_{p}{\rangle +\sin\alpha |↓}_{e}{↑}_{p}\rangle ,$$

the non-zero elements of the time-evolved density matrix ρ(t) can be analytically derived from the Lindblad master equation (see Appendix A). The density matrix at time *t* is found to be

(27) $$\rho \left(t\right)=\left(\begin{array}{cccc} \rho_{11}\left(t\right) & 0 & 0 & \rho_{14}\left(t\right)\\ 0 & \rho_{22}\left(t\right) & \rho_{23}\left(t\right) & 0\\ 0 & \rho_{32}\left(t\right) & \rho_{33}\left(t\right) & 0\\ \rho_{41}\left(t\right) & 0 & 0 & \rho_{44}\left(t\right) \end{array}\right)$$

where

(28) $$\begin{array}{cc} \rho_{11}\left(t\right) & =\frac{1}{4}\left[1-e^{-4\gamma t}\right],\\ \rho_{44}\left(t\right) & =\frac{1}{4}\left[1-e^{-4\gamma t}\right],\\ \rho_{22}\left(t\right) & =\frac{1}{4}\left[1+e^{-4\gamma t}+2\cos\left(2\alpha \right)\cos\left(4At\right)e^{-2\gamma t}\right],\\ \rho_{33}\left(t\right) & =\frac{1}{4}\left[1+e^{-4\gamma t}-2\cos\left(2\alpha \right)\cos\left(4At\right)e^{-2\gamma t}\right],\\ \rho_{23}\left(t\right) & =\frac{1}{2}e^{-2\gamma t}\left[i\cos(2\alpha )\sin(4At)+\sin(2\alpha )\right],\\ \rho_{ij}\left(t\right) & =0\;\mathrm{for}\;\mathrm{all}\;\mathrm{other}\;i,j, \end{array}$$

illustrating the decay of coherences and redistribution of populations over time. These solutions highlight the interplay between coherent oscillations (driven by *A*) and decoherence (governed by γ). The coherence between the states |1〉 and |4〉 decays exponentially with time, while the populations in the diagonal elements approach a uniform distribution due to the dissipative interactions.

**Figure 1:**
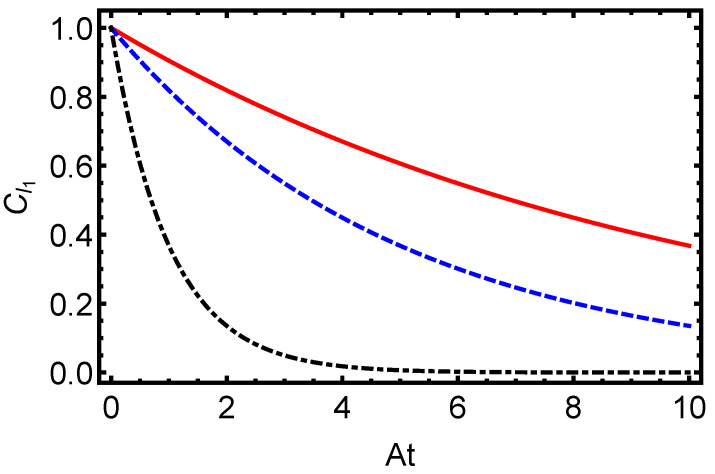
Time evolution of the $L_{1}$-norm of coherence for the electron–proton system in a hydrogen atom, initially prepared in a maximally entangled Bell state (α=π/4), under dissipative dynamics modeled by the Lindblad master equation. The plot illustrates the coherence decay for dissipation parameters γ=0.1 (solid line), γ=0.5 (dashed line), and γ=1.0 (dash-dotted line). The behavior of coherence highlights the detrimental effect of environmental interactions on QC in atomic systems, emphasizing the challenges of maintaining quantum properties under dissipation.

**Figure 2:**
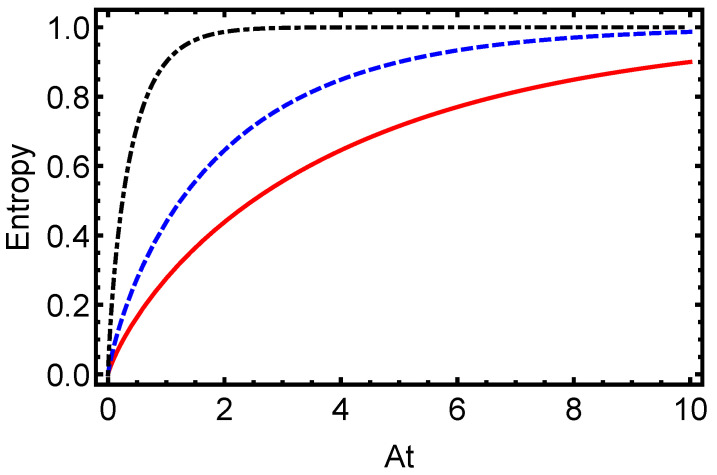
Time evolution of von Neumann entropy for the electron–proton state in a hydrogen atom, initially prepared in a maximally entangled Bell state (α=π/4), under dissipative dynamics modeled with the Lindblad master equation. The plot illustrates the entropy increase for varying dissipation parameters γ=0.1 (solid line), γ=0.5 (dashed line), and γ=1.0 (dash-dotted line). Each curve shows a rise from an initial zero entropy toward a maximum value. The rate of entropy growth accelerates with an increasing γ, underscoring the role of environmental dissipation in eroding quantum purity.

In Figure 1, we present the time evolution of the $L_{1}$-norm of coherence for the electron–proton system in a hydrogen atom, initially prepared in a maximally entangled Bell state (α=π/4), under dissipative dynamics modeled with the Lindblad master equation. This measure, defined as the sum of the absolute values of the off-diagonal elements of the density matrix, quantifies the QC in the system. The figure illustrates an exponential decay of the $L_{1}$-norm for dissipation parameters γ=0.1 (solid line), γ=0.5 (dashed line), and γ=1.0 (dash-dotted line), with the decay rate increasing as γ grows. This behavior underscores the rapid loss of quantum superpositions due to environmental interactions, highlighting the detrimental effect of dissipation on coherence in atomic systems. Complementing this, Figure 2 depicts the time evolution of the von Neumann entropy for the same electron–proton state under identical dissipative conditions. Starting from zero for the pure Bell state, the entropy increases toward a maximum value, indicating the system’s transition to a fully mixed state. The plot shows this rise for γ=0.1 (solid line), γ=0.5 (dashed line), and γ=1.0 (dash-dotted line), with the rate of entropy growth accelerating with higher γ. This trend emphasizes how stronger dissipation hastens the erosion of quantum purity and correlations. Together, Figure 1 and Figure 2 provide a comprehensive view of how dissipative dynamics undermine quantum features in hydrogen atoms. The exponential decay of the $L_{1}$-norm of coherence and the corresponding increase in von Neumann entropy reveal the challenges of maintaining quantum properties under environmental noise, critical for applications in quantum information technologies. By quantifying these effects, this study establishes a foundation for developing strategies to preserve coherence and purity in atomic systems against dissipation.

**Figure 3:**
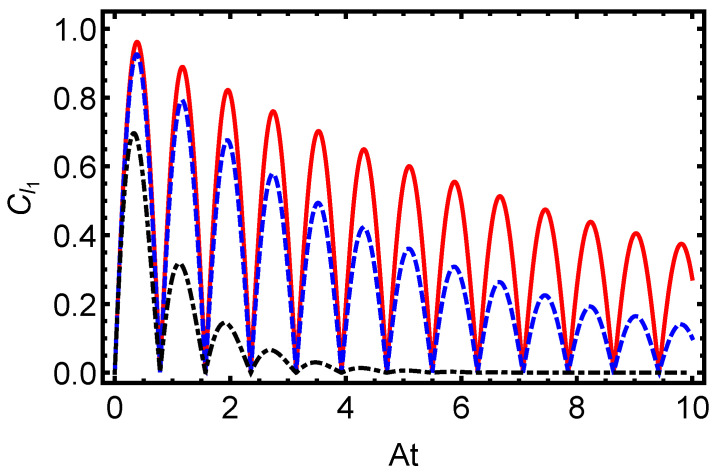
Time evolution of the $L_{1}$-norm of coherence for the electron–proton system in a hydrogen atom, initially prepared in a separable state (α=0), under dissipative dynamics governed by the Lindblad master equation. The plot illustrates the coherence dynamics for varying dissipation parameters γ=0.1 (solid line), γ=0.5 (dashed line), and γ=1.0 (dash-dotted line). Each curve displays oscillatory behavior driven by the hyperfine coupling constant, *A*, overlaid on an exponential decay envelope influenced by γ.

**Figure 4:**
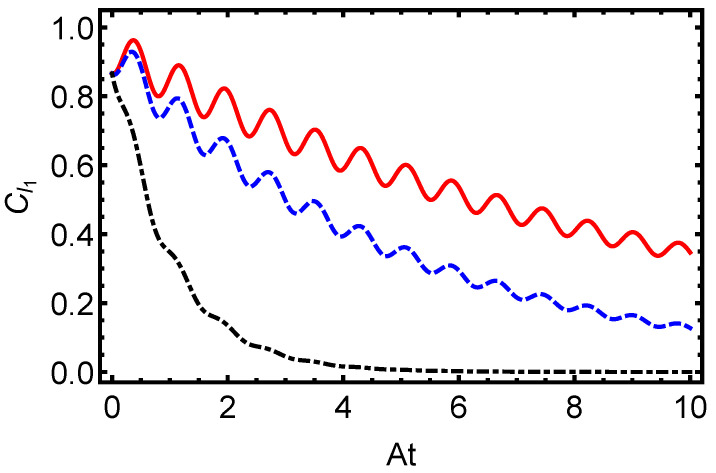
Time evolution of the $L_{1}$-norm of coherence for the electron–proton system in a hydrogen atom, initially in a partially entangled state (α=π/3), under dissipative dynamics governed by the Lindblad master equation. The coherence dynamics are shown for dissipation parameters γ=0.1 (solid line), γ=0.5 (dashed line), and γ=1.0 (dash-dotted line). The plot illustrates the coherence decay for dissipation parameters γ=0.1 (solid red line), γ=0.5 (dashed blue line), and γ=1.0 (dash-dotted black line). The behavior of coherence highlights the detrimental effect of environmental interactions on QC in atomic systems, emphasizing the challenges of maintaining quantum properties under dissipation.

In Figure 3, we present the time evolution of the $L_{1}$-norm of coherence for the electron–proton system in a hydrogen atom, initially prepared in a separable state (α=0), under dissipative dynamics governed by the Lindblad master equation. The figure illustrates oscillatory behavior driven by the hyperfine coupling constant *A*, overlaid with an exponential decay envelope governed by the dissipation parameter γ=0.1 (solid line), γ=0.5 (dashed line), and γ=1.0 (dash-dotted line). Higher values of γ accelerate the coherence loss, demonstrating the sensitivity of quantum superpositions to environmental interactions in atomic systems. Similarly, Figure 4 displays the time evolution of the $L_{1}$-norm of coherence for the electron–proton system in a hydrogen atom, initially prepared in a partially entangled state with α=π/3. The system evolves under dissipative dynamics governed by the Lindblad master equation, and the plot shows the effect of different dissipation rates.

**Figure 5:**
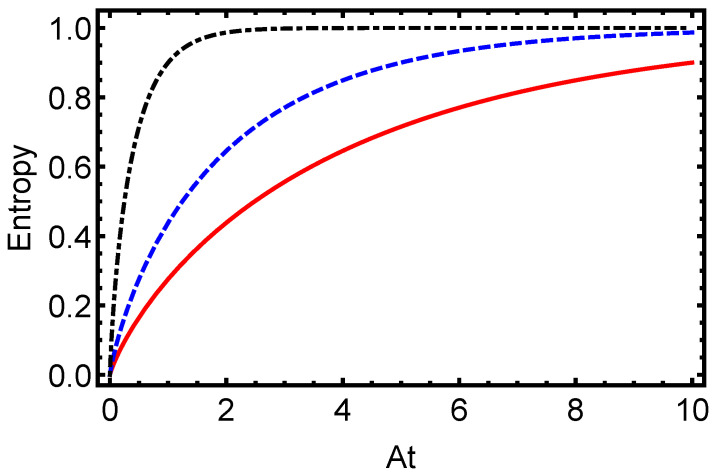
Time evolution of the von Neumann entropy for the electron–proton state in a hydrogen atom, initially prepared in a separable state (α=0), under dissipative dynamics governed by the Lindblad master equation. The plot illustrates the coherence dynamics for varying dissipation parameters γ=0.1 (solid line), γ=0.5 (dashed line), and γ=1.0 (dash-dotted line). Each curve displays oscillatory behavior driven by the hyperfine coupling constant *A*, overlaid on an exponential decay envelope influenced by γ.

The oscillatory pattern, modulated by *A*, is progressively suppressed by increasing γ, underscoring the detrimental impact of dissipation on quantum resources essential for applications such as quantum computing. Complementing these observations, Figure 5 depicts the time evolution of von Neumann entropy, an indicator of the system’s quantum purity. Starting from a low initial value for the separable state, the entropy rises steadily toward a maximum, signaling the system’s progression toward a more mixed state in the long-time limit, though not reaching the maximally mixed state, $I_{4}/4$, for which S(ρ)=ln(4)≈1.3863.The rate of this increase is directly tied to γ, with stronger dissipation hastening the loss of purity. Collectively, Figure 3, Figure 4 and Figure 5 illustrate how dissipative dynamics degrade key quantum features, coherence and purity, in hydrogen atoms. Despite the different initial states—with Figure 2 starting from a maximally entangled Bell state and Figure 5 from a separable state—the behavior of von Neumann entropy is remarkably similar in both cases. This similarity indicates that the entropy’s increase toward its maximum value, signaling the transition to a fully mixed state, is independent of the initial state of the system. Such behavior underscores the universal role of dissipation in eroding quantum purity, regardless of the system’s starting configuration. On the other hand, it is worth noting that coherence and mixedness are not independent quantities: for a given quantum state, ρ, the $l_{1}$ norm of coherence $C_{l_{1}}\left(\rho \right)$ and the linear mixedness $M_{l}\left(\rho \right)=\frac{d}{d-1}\left(1-\mathrm{Tr}\left[\rho^{2}\right]\right)$ satisfy a tradeoff relation, as discussed in [65,66]. This means that, as the system becomes more mixed (higher $M_{l}$), coherence tends to decrease, a behavior that is consistent with our numerical observations. 

## 4. Conclusions

In this paper, we have investigated the QC and purity in hydrogen atoms under dissipative dynamics, with a particular focus on the hyperfine structure states arising from electron–proton spin interactions. By employing the Lindblad master equation, we modeled the time evolution of the system’s density matrix, accounting for both the unitary dynamics driven by the hyperfine Hamiltonian and the dissipative effects due to environmental interactions. QC was quantified using the $L_{1}$-norm and relative entropy measures, while purity was assessed via von Neumann entropy. We examined two distinct initial states: a maximally entangled Bell state and a separable state. The results revealed distinct dynamical behaviors, depending on the initial state. For the Bell states, both coherence and purity decayed exponentially, with the decay rate directly proportional to the dissipation parameter γ. This underscored the significant impact of environmental interactions on quantum superpositions and correlations. In contrast, for certain separable states, coherence exhibited an oscillatory behavior modulated via the hyperfine coupling constant *A*, superimposed on exponential decay. This oscillatory pattern was accompanied by a steady increase in entropy, indicating a gradual loss of quantum purity. Furthermore, we observed that higher dissipation rates accelerated the loss of coherence and the growth of von Neumann entropy, highlighting the environment’s critical role in suppressing quantum superpositions and driving the system toward mixed states. These findings provided deeper insights into the preservation of coherence and purity in atomic systems, with important implications for quantum information applications that demand resilience against environmental noise. These results may offer practical insights for quantum information protocols, including robust state initialization, strategies for mitigating decoherence, and exploiting hyperfine interactions for encoding and manipulating qubits.

## Data Availability

The original contributions presented in this study are included in the article. Further inquiries can be directed to the corresponding author.

## Appendix A. Derivation of the Time-Evolved Density Matrix Elements

This appendix derives the analytic solution for the time-evolved density matrix ρ(t) of the hydrogen atom’s hyperfine structure, starting from the initial density matrix,

$$\rho \left(0\right)=\left(\begin{array}{cccc} 0 & 0 & 0 & 0\\ 0 & \cos^{2}\alpha & \cos\alpha \sin\alpha & 0\\ 0 & \cos\alpha \sin\alpha & \sin^{2}\alpha & 0\\ 0 & 0 & 0 & 0 \end{array}\right),$$

with non-zero elements $\rho_{22}\left(0\right)=\cos^{2}\alpha$, $\rho_{33}\left(0\right)=\sin^{2}\alpha$, $\rho_{23}\left(0\right)=\cos\alpha \sin\alpha$, and $\rho_{32}\left(0\right)=\cos\alpha \sin\alpha$. The Lindblad master equation yields a set of coupled differential equations for the density matrix elements,

(A1) $$\begin{array}{cc} \frac{d\rho_{11}}{dt} & =\gamma (\rho_{22}+\rho_{33}-2\rho_{11}), \end{array}$$

(A2) $$\begin{array}{cc} \frac{d\rho_{22}}{dt} & =-2iA\left(\rho_{23}-\rho_{32}\right)+\gamma \left(\rho_{11}+\rho_{44}-2\rho_{22}\right), \end{array}$$

(A3) $$\begin{array}{cc} \frac{d\rho_{33}}{dt} & =2iA\left(\rho_{23}-\rho_{32}\right)+\gamma \left(\rho_{11}+\rho_{44}-2\rho_{33}\right), \end{array}$$

(A4) $$\begin{array}{cc} \frac{d\rho_{44}}{dt} & =\gamma (\rho_{22}+\rho_{33}-2\rho_{44}), \end{array}$$

(A5) $$\begin{array}{cc} \frac{d\rho_{23}}{dt} & =-2iA\left(\rho_{22}-\rho_{33}+\rho_{23}\right)-2\gamma \rho_{23}, \end{array}$$

with $\rho_{32}\left(t\right)=\rho_{23}^{*}\left(t\right)$ due to the Hermitian property of ρ(t). Other off-diagonal elements, such as $\rho_{14}$, are found to remain zero under the given dynamics, consistent with the initial condition $\rho_{14}\left(0\right)=0$. To solve these equations, define $s\left(t\right)=\rho_{22}\left(t\right)+\rho_{33}\left(t\right)$. When Equations (A2) and (A3) are summed, the coherent terms cancel since ρ23−ρ32=2iIm(ρ23), yielding

$$\frac{ds}{dt}=2\gamma \left(\rho_{11}+\rho_{44}-s\right).$$

Since $\mathrm{Tr}\left[\rho \left(t\right)\right]=\rho_{11}+\rho_{22}+\rho_{33}+\rho_{44}=1$, we have $\rho_{11}+\rho_{44}=1-s$. Thus,

$$\frac{ds}{dt}=2\gamma \left(1-s-s\right)=2\gamma \left(1-2s\right).$$

Solving with the initial condition $s\left(0\right)=\cos^{2}\alpha +\sin^{2}\alpha =1$, we obtain the following:

$$s\left(t\right)=\frac{1}{2}+\frac{1}{2}e^{-4\gamma t}.$$

Since $\rho_{11}+\rho_{44}=1-s=\frac{1}{2}\left(1-e^{-4\gamma t}\right)$, and noting the symmetry in Equations (A1) and (A4) with $\rho_{11}\left(0\right)=\rho_{44}\left(0\right)=0$, we find

$$\rho_{11}\left(t\right)=\rho_{44}\left(t\right)=\frac{1}{4}\left(1-e^{-4\gamma t}\right).$$

Define $d\left(t\right)=\rho_{22}\left(t\right)-\rho_{33}\left(t\right)$ and $\rho_{23}\left(t\right)=c\left(t\right)=c_{r}\left(t\right)+ic_{i}\left(t\right)$. The system becomes the following:

(A6) $$\begin{array}{cc} \frac{dd}{dt} & =-2\gamma d-8Ac_{i}, \end{array}$$

(A7) $$\begin{array}{cc} \frac{dc_{r}}{dt} & =2Ac_{i}-2\gamma c_{r}, \end{array}$$

(A8) $$\begin{array}{cc} \frac{dc_{i}}{dt} & =-2A\left(d+c_{r}\right)-2\gamma c_{i}, \end{array}$$

with initial conditions $d\left(0\right)=\cos^{2}\alpha -\sin^{2}\alpha =\cos2\alpha$, $c_{r}\left(0\right)=\cos\alpha \sin\alpha =\frac{1}{2}\sin2\alpha$, and $c_{i}\left(0\right)=0$. Solving this coupled system analytically, we find that the coherent dynamics introduce oscillations at frequency 4A, modulated by dissipative decay. The solutions are

(A9) $$\begin{array}{cc} \rho_{22}\left(t\right) & =\frac{1}{4}\left[1+e^{-4\gamma t}+2\cos\left(2\alpha \right)\cos\left(4At\right)e^{-2\gamma t}\right], \end{array}$$

(A10) $$\begin{array}{cc} \rho_{33}\left(t\right) & =\frac{1}{4}\left[1+e^{-4\gamma t}-2\cos\left(2\alpha \right)\cos\left(4At\right)e^{-2\gamma t}\right], \end{array}$$

(A11) $$\begin{array}{cc} \rho_{23}\left(t\right) & =\frac{1}{2}e^{-2\gamma t}\left[i\cos(2\alpha )\sin(4At)+\sin(2\alpha )\right]. \end{array}$$

The conjugate $\rho_{32}\left(t\right)=\rho_{23}^{*}\left(t\right)=\frac{1}{2}e^{-2\gamma t}\left[-i\cos(2\alpha )\sin(4At)+\sin(2\alpha )\right]$.
